# Soft Tissue Augmentation After Tooth Extraction Improves Implant Health: Findings From a Clinical Trial

**DOI:** 10.7759/cureus.66263

**Published:** 2024-08-06

**Authors:** Ammar Ibrahim, Rowaida Saymeh, Basima Yosef

**Affiliations:** 1 Department of Periodontology, Faculty of Dental Medicine, Damascus University, Damascus, SYR; 2 Department of Histopathology, Faculty of Dentistry, Tishreen University, Latakia, SYR

**Keywords:** subepithelial connective tissue graft, xenogenic collagen matrix, dental implants, extraction socket, soft tissue augmentation

## Abstract

Background

Soft tissue augmentation is a critical procedure in dental implantology aimed at improving peri-implant health and aesthetics. Various materials are used for this purpose, but their comparative effectiveness remains under-researched. This study aimed to evaluate the effects of soft tissue augmentation utilizing two different materials after tooth extraction on peri-implant clinical and radiographic outcomes.

Methodology

A randomized controlled trial was conducted with 30 participants requiring extraction of non-restorable mandibular posterior teeth. Participants were randomly assigned to receive connective tissue graft (CTG), Fibro-gide (FG), or spontaneous healing (SH) in a 1:1:1 allocation ratio. Two months post-treatment, dental implants were placed. Six months after the functional loading of the dental implant, peri-implant health was assessed using the Plaque Accumulation Index, bleeding on probing (BOP), pocket depth, mucosal recession, and marginal bone level.

Results

At the six-month follow-up, the SH group exhibited significantly higher Plaque Index and BOP percentages (6.43 ± 1.23 and 70%, respectively) compared to the CTG group (0.40 ± 0.32 and 8.3%, respectively) and FG group (0.45 ± 0.44 and 9.7%, respectively). The mean probing pocket depth was also significantly higher in the control group (5.13 ± 0.64 mm), while the CTG and FG groups showed minimal changes (3.83 ± 0.39 mm for both groups). Additionally, gingival recession was higher in the control group (0.65 ± 0.18 mm) compared to the CTG and FG groups (0.03 ± 0.08 mm for both groups). Radiographic analysis revealed greater marginal bone loss in the control group (0.40 ± 0.05 mm) compared to the CTG and FG groups, which demonstrated minimal bone loss (0.17 ± 0.08 mm and 0.20 ± 0.00 mm, respectively).

Conclusions

The study findings indicate that FG is as effective as CTG in maintaining peri-implant health, outperforming SH. These findings suggest that FG can be a viable alternative to CTG in soft tissue augmentation after tooth extraction, offering a new option for clinicians in the management of extraction sites before dental implant placement.

## Introduction

The width of keratinized tissue (KT) surrounding natural teeth does not appear to be linked to periodontal health maintenance. Various studies have indicated that similar levels of plaque accumulation, gingival inflammation, and periodontal attachment maintenance are observed regardless of KT width, provided that adequate oral hygiene is maintained [1]. A recent long-term study found that, over a follow-up period of 10 to 27 years, sites with a narrow band of KT were more likely to develop gingival recession (GR) and tissue inflammation compared to sites that had been surgically augmented [2]. When considering peri-implant tissues, it is important to note that anatomical differences between periodontal and peri-implant tissues complicate the interpretation of clinical outcomes. An attachment forms between the implant abutment surface and the adjacent epithelium, characterized by glycoproteins similar to those found between the epithelium and natural tooth surfaces [3]. In natural teeth, some gingival fibers run perpendicularly to the root surfaces and insert into the root cementum. In contrast, all peri-implant connective tissue fibers run parallel or obliquely to the titanium surfaces and do not attach to the implant surface [4].

Autogenous soft tissue grafting procedures have been suggested for surgically correcting localized alveolar defects, pre-prosthetic site development, and ridge preservation [5]. For augmenting KT, the free gingival graft and the subepithelial connective tissue graft (CTG) have traditionally been used to increase soft tissue volume [6,7]. The main disadvantages of using autogenous tissue stem from the harvesting procedure, which results in prolonged healing time at the donor site and increased patient morbidity. Patients often report pain and numbness for several weeks post-surgery [8]. Additionally, there are anatomical and individual limitations. The quantity and quality of tissue that can be harvested vary depending on the shape of the palatal vault and the patient’s sex and age. The location of palatal vessels and nerves further restricts the total amount available for grafting procedures [9].

In response to these challenges, a cross-linked, porcine-derived collagen matrix (Geistlich Fibro-Gide® (GF)) has been introduced as an innovative alternative. This material aims to replicate the benefits of CTGs while minimizing potential complications associated with autogenous grafts. A pre-clinical study by Herford et al. [10] and a clinical study by De Angelis et al. [11] have both evaluated the efficacy of this novel material. Their findings suggest that the collagen matrix not only matches the performance of CTGs in terms of soft tissue augmentation but also offers additional benefits. These include decreased postoperative pain, shorter surgical time, and enhanced patient satisfaction. However, it is important to note that the studies’ conclusions were tempered by certain methodological shortcomings, such as the absence of randomization and a lack of sample size calculation, which could affect the validity of the results.

This study aimed to evaluate the effects of soft tissue augmentation utilizing two different materials (CTG, FG, and spontaneous healing (SH) without soft tissue augmentation) after tooth extraction on peri-implant clinical and radiographic outcomes.

## Materials and methods

Study design and sample size calculation

**Figure 1:**
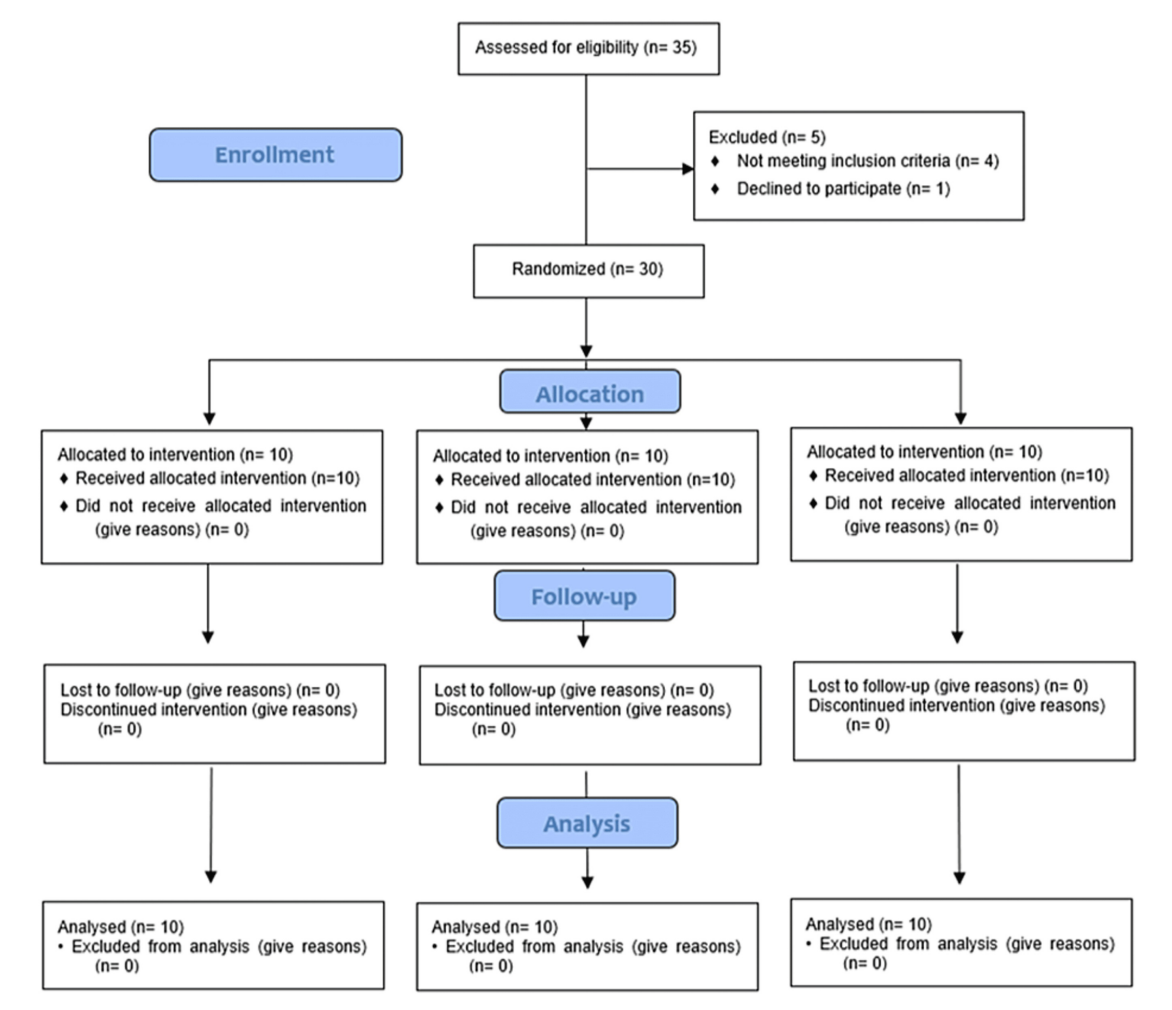
Consolidated Standards of Reporting Trials (CONSORT) 2010 flow diagram.

This randomized controlled trial aimed to evaluate the effects of soft tissue augmentation using two different materials (CTG and xenogenic collagen matrix) and SH without soft tissue augmentation on peri-implant clinical and radiographic outcomes after tooth extraction. In total, 30 participants aged between 25 and 46 were selected for the extraction of 30 non-restorable mandibular posterior teeth, which exhibited no periodontal disease. The mandibular posterior teeth were chosen due to their high functional load and common occurrence of non-restorable conditions, making them ideal candidates for evaluating the effectiveness of soft tissue augmentation. The age range of 25 to 46 years was selected to include a population with sufficient bone density and healing capacity, while also representing a typical demographic for dental implant procedures. Following extraction, subjects were randomly assigned to one of three treatments, i.e., CTG, FG, or SH, with an equal distribution among the groups (allocation ratio 1:1:1). Two months after these interventions, dental implants were placed. The research was conducted at the Department of Periodontology, Faculty of Dentistry, Damascus University, adhering to the ethical standards outlined in the 2000 revision of the Helsinki Declaration. Comprehensive information regarding the study’s procedures, objectives, and potential risks was provided to all qualifying participants, who then gave their written informed consent. The study’s protocol received ethical clearance from the Damascus University Ethics Committee (approval number: UDDS‐28066021/SRC‐2486), and the reporting was guided by the Consolidated Standards of Reporting Trials (CONSORT) statement checklist (Figure 1).

This study was registered at the ISRCTN registry (study ID: ISRCTN18692174). To calculate the necessary sample size, the G*power software (version 3.1.9.7) was utilized. A total of 21 individuals (seven per treatment group) were required to ensure a 5% Type I error rate and an 80% statistical power. The anticipated effect size of 1.73 was derived from a projected 10% difference in bone width among the groups, with the largest reported standard deviation being 1.62 mm [12]. Considering an estimated dropout rate of 20%, the study included 30 patients, with 10 in each group.

Inclusion and exclusion criteria

Participants were included in the study if they met the following conditions: had extraction sites that conformed to Type ST1 (Socket type 1) classification, as per Steigmann et al. [13], where both facial soft and hard tissues were preserved at levels consistent with the cementoenamel junction; possessed at least 2 mm of KT on the buccal aspect of the extraction site; demonstrated commendable oral hygiene; and were aged 18 years or above.

The study excluded individuals who exhibited buccal alveolar bone defects, such as dehiscence or fenestration, or experienced loss of the facial bone plate during extraction. Patients with systemic conditions that could impede bone healing were also excluded. Individuals who were pregnant at the time of the study and smokers were also excluded.

Surgical procedure

**Figure 2:**
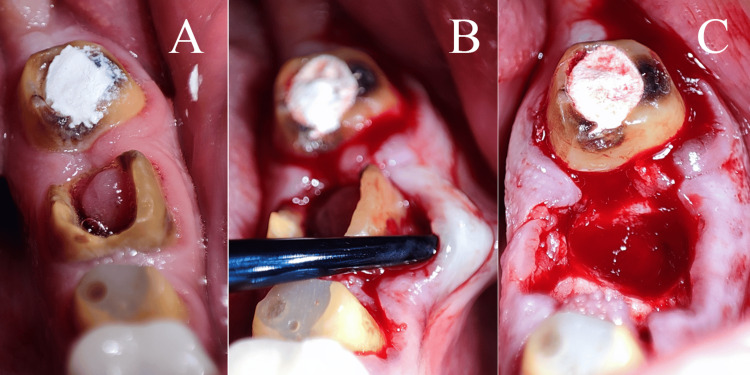
Atraumatic tooth extraction.

Before the surgical intervention, all patients underwent clinical and radiographic evaluations. As a preoperative measure, patients were instructed to rinse with povidone-iodine mouthwash for one minute. Local anesthesia was administered at the site of the procedure using 2% lidocaine with 1:80,000 epinephrine (Kwang Myung Pharm, Sindaebang 1-dong Dongjak-gu, Seoul, Korea). Prophylactic antibiotics were given one hour before the surgery (2 g of amoxicillin or, for those with penicillin allergies, 600 mg of clindamycin). Atraumatic tooth extractions were performed (Figure 2) using an Atraumatic Extraction Kit, Black Line (IMNATREXTX, HuFriedyGroup, Chicago, USA), followed by thorough curettage of the extraction sockets to remove any residual soft tissue. At this juncture, a sealed envelope was opened to assign each patient to one of the three study groups, i.e., SH, CTG, or FG.

**Figure 3:**
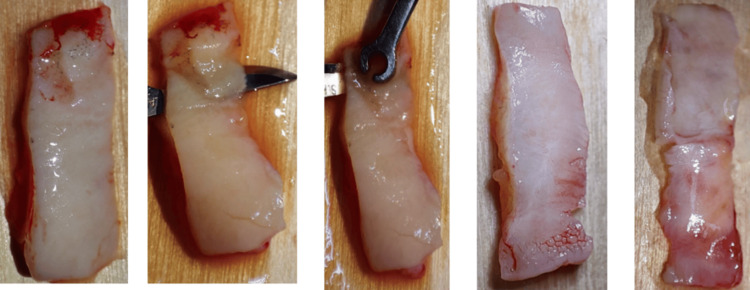
The harvested connective tissue graft de-epithelization technique.

In the SH group, extraction sockets were left to heal naturally without intervention. In the CTG group, soft tissue augmentation was done using CTG alone. The technique involved elevating a partial-thickness flap; dissection was extended horizontally to the mucogingival junction at the extraction site while preserving the interdental papillae and avoiding extension to adjacent teeth. Raj et al. [14] detailed the use of a barrier material to seal the socket post-extraction, thereby preserving the alveolar bone integrity. Subsequently, a CTG was harvested from the palate following the method described by Zucchelli et al. [15] (Figure 3).

**Figure 4:**
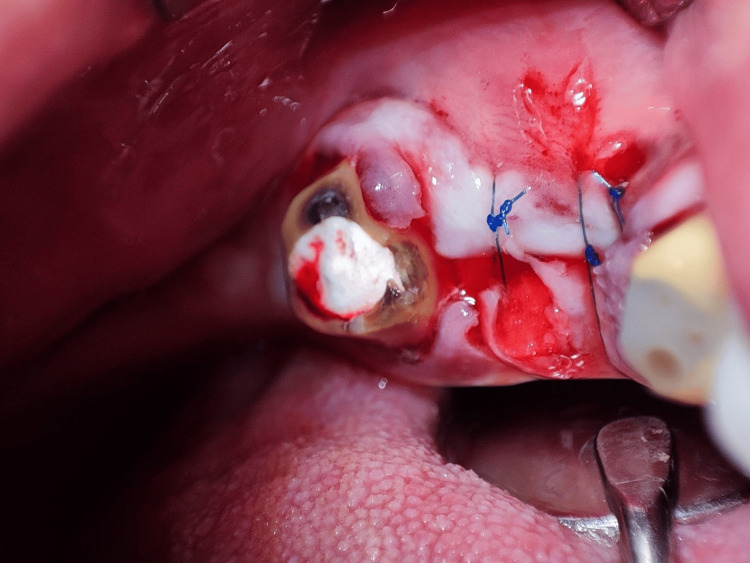
The coronal repositioning of the flap to secure the graft.

The graft was then sutured into place using 6/0 monofilament polypropylene non-absorbable suture material (Vertpro, VertMed GmbH, Germany), and the flap was coronally repositioned to secure the graft (Figure 4).

**Figure 5:**
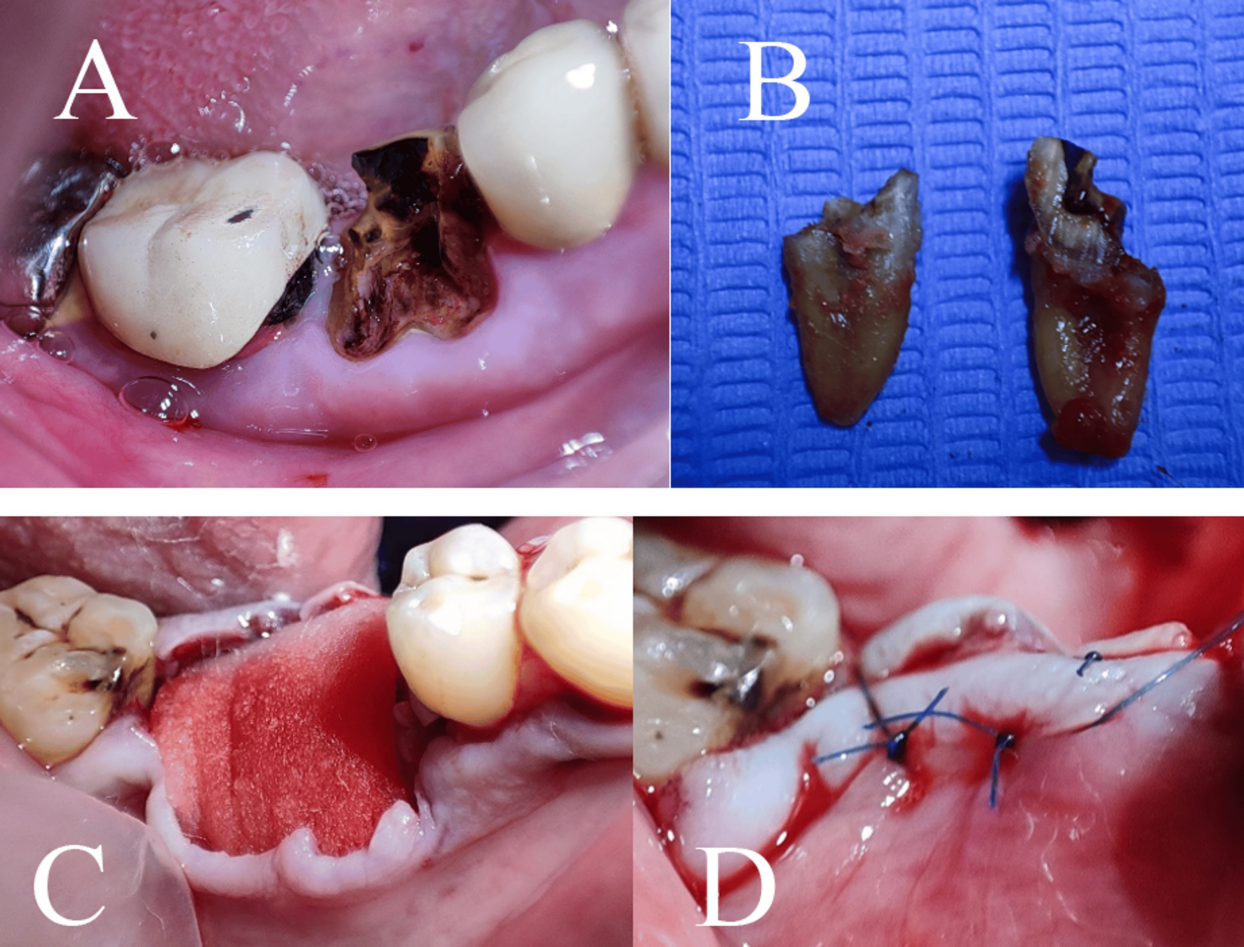
The surgical procedure in the Fibro-Gide® group: hopeless tooth (A), extraction of tooth (B), application of xenogeneic collagen matrix (C), and flap suturing (D).

In the FG group, Fibro-Gide® collagen matrix replaced CTG as used in the second group. The matrix was tailored to fit the dimensions of the extraction site and applied similarly to the CTG (Figure 5).

Postoperatively, all patients received 500 mg of amoxicillin three times daily for eight days and were advised to use 0.12% chlorhexidine mouthwash twice daily for three weeks.

**Figure 6:**
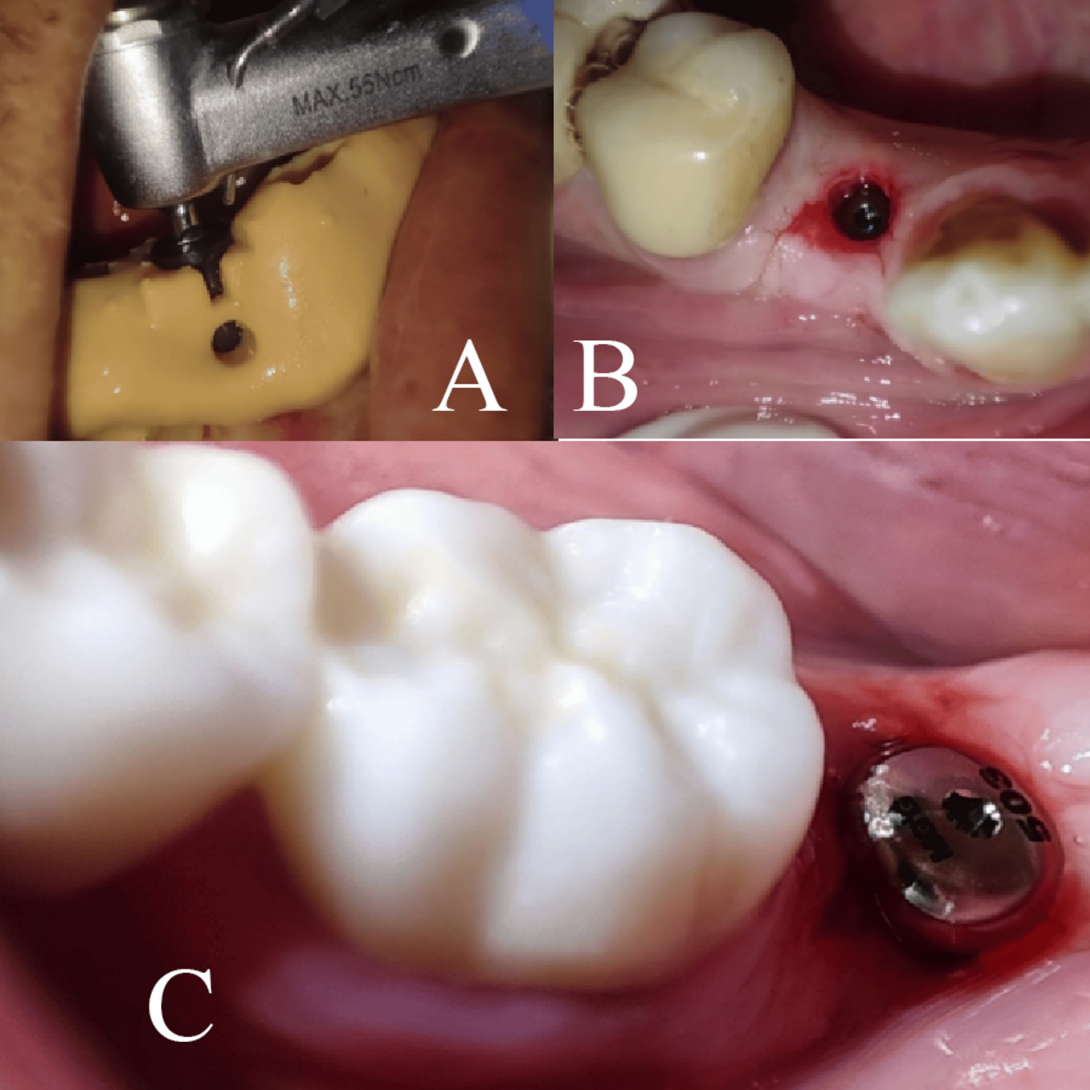
Implant placement two months after the procedure: guided surgery (A), implant placement (B), and healing abutment (C).

Two months following the procedures, AnyRidge® implants (MegaGen Implant Co., Ltd., Daegu, South Korea) were placed at the sites of extracted teeth using a three-dimensional-printed surgical guide in a flapless manner. Healing abutments were attached to all implants immediately after placement (Figure 6). Patients received postoperative instructions regarding diet and medication management.

Outcome measurements

At each implant location, a comprehensive assessment of periodontal health was conducted utilizing a calibrated probe. This evaluation encompassed several metrics: (1) Plaque Accumulation Index (PAI), adapted from the methodology proposed by Löe and colleagues [16]; (2) bleeding on probing (BOP), a binary assessment indicating the presence or absence of bleeding; (3) pocket depth (PD), gauged from the edge of the mucosa to the base of the pocket; and (4) mucosal recession (MR), the distance from the edge of the restoration to the gingival margin. These parameters, i.e., PAI, BOP, PD, and MR, were meticulously measured at the following six distinct points around each implant to ensure a thorough evaluation: mesiobuccal (mb), buccal (b), distobuccal (db), mesio-oral (mo), oral (o), and disto-oral (do).

The evaluation of marginal bone levels was conducted at two key intervals: initially, at the time of implant insertion (baseline), and, subsequently, after six months. For each dental implant, standardized periapical radiographs were captured at baseline and the six-month mark, employing a paralleling apparatus (Dentsply Rinn, Rinn Corporation, Elgin, IL, USA) alongside a standard paralleling method (Kavo In Exam, dental X-ray unit, 70 kVp, 7 Ma, 0.115 seconds). The quantification of marginal bone loss (MBL) involved measuring the span from the inaugural point of bone contact with the implant to the crest of the implant. The assessment of bone loss incorporated both the mesial and distal dimensions of each dental implant, with the resultant figures being amalgamated to ascertain the average bone loss. All radiographic exposures and subsequent measurements were consistently executed by a single clinician who was not privy to the allocation of study groups. Cone-beam CT was avoided due to its higher radiation dose compared to periapical radiographs, as well as the increased cost and limited accessibility in some clinical settings. Standardized periapical radiographs were deemed sufficient for the accurate measurement of MBL in this study.

Statistical analysis

All statistical analyses were conducted using SPSS software version 29.0.2.0 (IBM Corp., Armonk, NY, USA). The Kolmogorov-Smirnov test was employed to evaluate the normality of the parameters. A p-value less than 0.05 was considered statistically significant. Categorical data were represented as percentages, while continuous variables were described using the mean and standard deviation. To discern the differences in bleeding on probing across various groups, the chi-square test was utilized. The Kruskal-Wallis test facilitated comparisons among groups concerning the Plaque Index (PI), PD, MR, and marginal bone level. Differences between the periods under study were determined using the Mann-Whitney U test and the Wilcoxon test.

## Results

All participants successfully completed the study. Figure 1 illustrates the flow of participants throughout the study’s duration. Healing at the sites for the CTG, FG, and SH group occurred without complications.

Demographic data

The average age of participants in the CTG group was 34.1 ± 5.9 years. The FG group had a slightly higher average age of 34.9 ± 6.1 years, and the control group had the highest average age of 35.6 ± 6.4 years (Table 1). The FG group comprised a larger proportion of males (70.0%) compared to the CTG (50.0%) and control groups (50.0%). Despite these variations, no significant demographic differences were found between the groups. All interventions were performed in the posterior lower arch, with the CTG group having three premolars and seven molars, the FG group having two premolars and eight molars, and the control group having four premolars and six molars.

**Table 1:** Demographic characteristics of the included participants in each group. CTG = connective tissue graft; FG = Fibro-Gide

Characteristics	Group, mean (SD) or N (%)			P-value
	CTG (n = 10)	FG (n = 10)	Control (n = 10)	
Age	34.1 (5.9)	34.9 (6.1)	35.6 (6.4)	0.149
Sex				
Male	5 (50%)	7 (50%)	5 (50%)	0.337
Female	5 (50%)	3 (30%)	5 (50%)	
Extracted tooth location				
Premolars	3 (30%)	2 (20%)	4 (40%)	0.371
Molars	7 (70%)	8 (80%)	6 (60%)	0.459

Clinical parameters

Table 2 displays the clinical parameters for the three groups.

**Table 2:** Clinical parameters of the groups and the results of chi-square and Kruskal-Wallis tests. *: Statistically significant differences. BOP = bleeding on probing; CTG = connective tissue graft; GR = gingival recession; FG = Fibro-Gide; PI = Plaque Index; PPD = peri-implant probing depth

	Baseline					Sixth months				
	CTG	FG	Control	Chi-square value	P-value	CTG	FG	Control	Chi-square value	P-value
PI	0.16 ± 0.29	0.18 ± 0.30	0.18 ± 0.30	0	1.000	0.40 ± 0.32	0.45 ± 0.44	6.43 ± 1.23	15.904	0.001*
BOP (%)	6.7%	7.4%	8.5%	0.381	0.537	8.3%	9.7%	70%	4.444	0.035*
PPD	3.67 ± 0.49	3.75 ± 0.45	3.86 ± 0.41	2.540	0.660	3.83 ± 0.39	3.83 ± 0.39	5.13 ± 0.64	19.672	0.000*
GR	-					0.03 ± 0.08	0.03 ± 0.08	0.65 ± 0.18	23.165	0.000*

Plaque Index

At baseline, the groups showed no significant differences in PI (p = 1.000). However, at the six-month follow-up, the control group exhibited a significantly higher PI (p = 0.001) than the CTG and FG groups. Longitudinal analysis within the control group indicated an increase in plaque accumulation over time, with significant differences noted between baseline and six months (p = 0.009). The CTG and FG groups did not show such changes (p = 0.083 for both).

Bleeding on Probing

Initially, no BOP was observed in any group (p > 0.05). At six months, significant differences emerged, with the control group showing a higher percentage of BOP (70.0%) compared to the CTG (8.3%) and FG (9.7%) groups. The control group’s BOP percentage increased significantly from baseline to six months (p = 0.007), unlike the CTG and FG groups (p = 1.000 for both).

Probing Pocket Depth

No significant differences in PPD were noted at baseline (p > 0.05). After six months, the control group’s mean PPD was significantly higher (5.13 ± 0.64 mm) than that of the CTG and FG groups (3.83 ± 0.39 mm for both). Within-group comparisons revealed a slight, non-significant increase in PPD in the CTG and FG groups from baseline to six months (p > 0.05). Conversely, the control group experienced a significant increase in PPD over the same period (p < 0.05).

Gingival Recession

Significant differences in GR were observed among the groups (p < 0.05). The CTG and FG groups had a minimal mean recession of 0.03 ± 0.08 mm, while the control group showed a greater mean recession of 0.65 ± 0.18 mm.

Radiographic analysis

At baseline, the marginal bone level was determined, revealing no significant differences between groups (p > 0.05). Upon reassessment after six months, significant differences were noted (p < 0.05), with the control group exhibiting greater MBL than the other groups. Table 3 provides further details on MBL. A significant increase in MBL was observed in all groups after six months (p < 0.05) compared to baseline.

**Table 3:** Mean MBL among groups and the results of the Kruskal-Wallis test. *: Statistically significant differences CTG = connective tissue graft; FG = Fibro-Gide; MBL = marginal bone level; SD = standard deviation

Group	Baseline			Sixth month			MBL (sixth month – baseline)
	Mean ± SD	Chi-square value	P-value	Mean ± SD	Chi-square value	P-value	
CTG	-0.15 ± 0.05	4.209	0.418	-0.32 ± 0.11	13.489	0.002*	0.17 ± 0.08
FG	-0.13 ± 0.05			-0.33 ± 0.05			0.20 ± 0.00
Control	-0.17 ± 0.08			-0.67 ± 0.19			0.40 ± 0.05

## Discussion

This randomized controlled trial provides compelling evidence for the efficacy of FG in soft tissue augmentation, demonstrating its comparable performance to traditional CTGs and superiority over SH. The study’s findings are significant in the context of dental implantology, as they suggest that FG can be a viable alternative to CTGs, offering clinicians more options for treatment. A narrative review by Kim and Kim in 2024 highlighted the advancements in the form of soft tissue augmentation, emphasizing the shift toward innovative materials and techniques. These approaches have been shown to accelerate bone healing and improve patient-reported outcomes, including satisfaction and quality of life. The review also noted a high implant survival rate over five to seven years, indicating the reliability of these new augmentation methods [17]. The initial uniformity in PI across all groups indicates a consistent baseline for comparison. The significant increase in PI within the control group at the six-month follow-up suggests that SH may not be effective in maintaining plaque control compared to the intervention groups. The stability of PI in the CTG and FG groups underscores the potential of these treatments in preserving periodontal health post-extraction, which is crucial for the long-term success of dental implants [18]. The absence of BOP at baseline across all groups reflects the exclusion of participants with periodontal disease, ensuring that the study’s focus remains on the efficacy of the soft tissue augmentation methods. The marked increase in BOP in the control group at six months highlights the importance of intervention in preventing inflammatory responses post-extraction [19]. The low incidence of BOP in the CTG and FG groups is indicative of their protective effects against gingival inflammation. The lack of significant differences in PD at baseline provided a level playing field for assessing the impact of the different treatments. The significant increase in PD in the control group suggests that SH may lead to suboptimal conditions for implant placement, potentially compromising implant stability. The negligible changes in PD within the CTG and FG groups not only affirm their effectiveness in maintaining tissue architecture but also suggest their role in facilitating optimal conditions for subsequent implant integration. The minimal GR observed in the CTG and FG groups is a positive outcome, indicating that both treatments can effectively preserve the mucosal margin. In contrast, the greater recession in the control group points to the potential for aesthetic and functional deficits associated with SH, which could affect the prognosis of dental implants. The radiographic findings corroborate the clinical measurements, with the control group exhibiting greater MBL compared to the intervention groups. This radiographic evidence further supports the notion that FG and CTG can maintain alveolar ridge integrity, which is essential for the long-term stability of dental implants [20]. Our findings are comparable to the findings of a systematic review and meta-analysis focused on the association of CTGs with immediate implants. The study concluded that CTGs associated with immediate implant placement can maintain gingival levels but not increase the volume, which is favorable for achieving successful aesthetic results [21]. Our findings are also consistent with previous studies that reported the advantages of using CTGs over other materials for ridge preservation [22]. However, the FG group showed acceptable outcomes, especially in terms of gingival thickness, which is an important factor regarding the esthetic outcomes of dental implants.

In comparing the histological superiority of FG to CTG, it is important to note that FG has demonstrated significant advantages in promoting soft tissue formation. An in vitro study evaluating two tissue substitutes for gingival augmentation found that FG induced a higher secretion of type I collagen, matrix metalloproteinase-2, tissue inhibitor of metalloproteinases (TIMP)-1, and TIMP-2 compared to Mucoderm®, another collagen matrix. These proteins are crucial for tissue regeneration and stability, suggesting that FG provides a more conducive environment for soft tissue integration. Additionally, FG’s three-dimensional structure supports angiogenesis and the formation of new connective tissue, which are essential for successful grafting. The study also revealed that FG exhibited a lower surface roughness compared to Mucoderm®, which may contribute to its superior performance in soft tissue augmentation. This evidence underscores FG’s potential as a superior alternative to CTG, offering similar or enhanced outcomes without the need for a second surgical site, thereby reducing patient morbidity and improving overall treatment efficiency [23].

One limitation of this study is the relatively short follow-up period of six months, which may not fully capture the long-term outcomes and potential complications associated with soft tissue augmentation using FG. Additionally, the study’s sample size, while adequate for initial comparisons, may limit the generalizability of the findings to a broader population. The exclusion of participants with periodontal disease, while necessary for focusing on the efficacy of the preservation methods, may also limit the applicability of the results to patients with varying periodontal health statuses. Furthermore, the study did not account for potential confounding factors such as variations in surgical technique and patient compliance with postoperative care, which could influence the outcomes. The advantages of CTG include proven efficacy, biocompatibility, and good integration with existing tissues, but it requires a second surgical site, increases patient morbidity, and has a longer recovery time. FG eliminates the need for a second surgical site, reduces patient morbidity, and is easy to use, but it has limited long-term data, potential variability in outcomes, and higher cost compared to CTG. SH is cost-effective and requires no additional materials or procedures, but it has less predictable outcomes, a higher risk of complications, and the potential for inadequate soft tissue volume. Considering the small sample size, it is possible to summon patients for a longer follow-up to improve the quality of the study. According to periodontics literature, a follow-up period of at least 12 months is recommended to evaluate the success of soft tissue augmentation treatments comprehensively. Extending the follow-up period would provide more robust data on the long-term stability and effectiveness of the materials used.

## Conclusions

This study highlights the potential of FG as an effective material for soft tissue augmentation, demonstrating comparable results to CTGs and superior outcomes to SH. Histologically, FG has been shown to support angiogenesis, the formation of new connective tissue, and the stability of the collagen network, which are critical for successful soft tissue integration. Studies have indicated that FG provides volume stability and promotes soft tissue regeneration without the need for a second surgical site, reducing patient morbidity and treatment time. These findings suggest that FG can offer clinicians a viable alternative to traditional methods, enhancing treatment options in dental implantology. The study underscores the importance of innovative materials in improving patient outcomes and provides a foundation for future research to explore long-term effects and broader clinical applications of FG in various dental scenarios. These insights contribute to advancing clinical practices and optimizing patient care in the field of dental surgery.
